# Lipopolysaccharide structure impacts the entry kinetics of bacterial outer membrane vesicles into host cells

**DOI:** 10.1371/journal.ppat.1006760

**Published:** 2017-11-29

**Authors:** Eloise J. O’Donoghue, Natalie Sirisaengtaksin, Douglas F. Browning, Ewa Bielska, Mohammed Hadis, Francisco Fernandez-Trillo, Luke Alderwick, Sara Jabbari, Anne Marie Krachler

**Affiliations:** 1 Institute of Microbiology and Infection, School of Biosciences, University of Birmingham, Edgbaston, Birmingham, United Kingdom; 2 Department of Microbiology and Molecular Genetics, University of Texas McGovern Medical School at Houston, Houston, Texas, United States of America; 3 Institute of Microbiology and Infection, School of Chemistry, University of Birmingham, Edgbaston, Birmingham, United Kingdom; 4 School of Mathematics, University of Birmingham, Edgbaston, Birmingham, United Kingdom; The University of Texas at Austin, UNITED STATES

## Abstract

Outer membrane vesicles are nano-sized microvesicles shed from the outer membrane of Gram-negative bacteria and play important roles in immune priming and disease pathogenesis. However, our current mechanistic understanding of vesicle-host cell interactions is limited by a lack of methods to study the rapid kinetics of vesicle entry and cargo delivery to host cells. Here, we describe a highly sensitive method to study the kinetics of vesicle entry into host cells in real-time using a genetically encoded, vesicle-targeted probe. We found that the route of vesicular uptake, and thus entry kinetics and efficiency, are shaped by bacterial cell wall composition. The presence of lipopolysaccharide O antigen enables vesicles to bypass clathrin-mediated endocytosis, which enhances both their entry rate and efficiency into host cells. Collectively, our findings highlight the composition of the bacterial cell wall as a major determinant of secretion-independent delivery of virulence factors during Gram-negative infections.

## Author summary

All Gram negative species of bacteria, including those that cause significant disease, release small vesicles from their cell membrane. These vesicles deliver toxins and other virulence factors to host cells during infection. Current methods for studying host cell entry are limited due to the nanometer size and rapid uptake kinetics of vesicles. Here we developed a method to monitor the rapid vesicle entry into host cells in real-time. This method highlighted differences in kinetics and entry route of vesicles into host cells, which varied with the bacterial cell wall composition and thus, the vesicle surface. Increased understanding of vesicular entry mechanisms could identify targets which may allow us to combat infections by inhibiting delivery of vesicle-associated toxins to host cells.

## Introduction

Outer membrane vesicles (OMVs) are nano-sized proteoliposomes released from the bacterial cell envelope [1]. OMV release is a highly conserved process, occurring in all growth phases and environmental conditions [2]. OMVs contain and deliver a broad range of cargos, from large hydrophobic molecules to DNA, making them a versatile and generalised form of secretion that enhances bacterial fitness in hostile environments [3–6]. They also contribute significantly to pathogenesis, via the delivery of virulence factors such as toxins, adhesins and immunomodulatory compounds directly into the host cell [7–9]. In a mouse model, purified OMVs from *Escherichia coli* were sufficient to cause lethal sepsis in the absence of intact bacterial cells, indicating their potency in enhancing infection and inflammatory processes [10]. The immunogenicity and ubiquitous production of OMVs has also led to their clinical use in vaccine preparations [11], representing an application for OMVs in generating immunity against bacterial infections without the risks associated with live cell vaccines. Whilst many virulence factors are known to be OMV cargos, the processes underlying their delivery to host cells during infection are not well characterized. Understanding these mechanisms could help to identify targets for inhibition of OMV-associated toxin delivery and lead to attenuation of bacterial infections, as well as helping to achieve their therapeutic potential in medicine, via vaccines and engineered delivery vehicles [12–14].

Release of OMVs occurs during infection, and has advantages over other secretion systems. They can carry a broad range of cargos, from protein toxins to hydrophobic small molecules such as the *Pseudomonas aeruginosa* quorum sensing molecule quinolone signal (PQS), and vesicular cargos are protected from environmental insults [15, 16]. In addition, OMV-mediated delivery of virulence factors can function over longer distances than contact-dependent secretory pathways [17]. While much is known about the cargos contained within OMVs, the small size of OMVs (20–200 nm) and rapid kinetics of entry (cargo-specific effects can often be detected within minutes) have made studying their interactions with host cells difficult. Previous work has often relied on OMVs labelled with dyes, non-discriminate probes that modify vesicular contents during labeling. While such probes allow real-time analysis of OMV entry and cargo delivery, their potential to modify vesicle components may interfere with the vesicle’s physicochemical characteristics, and alter the mechanism of OMV recognition, entry and cargo release [18–20]. Other approaches rely on immunolabelling of OMV-associated epitopes, but this often requires fixation of cells at pre-determined time points, and makes assumptions about OMV cargo, which may ignore natural sub-populations of OMVs [21]. Some experiments have used specific changes in host cell phenotypes in response to OMV contained toxins as an indicator of OMV uptake [4]. However, such changes in host cell responses have distinct dynamics from the OMV entry event, and allow only indirect conclusions about entry kinetics [22]. These challenges have often led to discrepancies in observations of OMV entry and cargo delivery [14], demonstrating the need for an assay that can detect OMV entry processes in a consistent and repeatable manner. In this paper we describe a novel assay to continuously measure OMV entry and cargo release to host cells with high sensitivity, and in a format that is adaptable for high throughput screening. Using this assay to study entry of OMVs from different *E*. *coli* serotypes and pathovars into host cells, we identified key bacterial and host factors that determine the route of entry, and thereby kinetics and efficiency of vesicular cargo delivery and trafficking.

## Results

### A highly sensitive, kinetic assay for monitoring OMV entry into host cells

**Fig 1:**
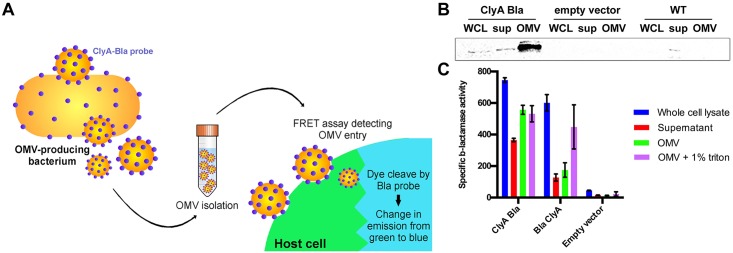
Genetically encoded Bla probes are enriched in *E*. *coli* OMVs and retain their enzymatic activity. (A) Expression of genetically encoded Bla probes is induced in bacteria and secreted OMVs are isolated for all subsequent experiments. Entry of OMVs containing Bla probes into host cells can be detected using a continuous FRET assay. (B) Whole cell lysate (WCL), supernatant (sup) and outer membrane vesicles (OMV) fractions isolated from EHEC expressing ClyA-Bla, carrying empty vector, or no vector were separated by SDS-PAGE and expression of ClyA-Bla was detected by Western Blotting and probing with α-Bla antibody. (C) Specific enzyme activity in whole cell lysate, supernatant, OMV or solubilized OMV fractions isolated from EHEC expressing ClyA-Bla, Bla-ClyA, or carrying empty vector (data shown are means ± stdev, n = 3).

We set out to develop a highly sensitive and dynamic assay that would allow us to monitor the kinetics of OMV entry into host cells. We used a genetically encoded hybrid reporter probe that is incorporated into the bacterial outer membrane and subsequently targeted to the OMV surface. ClyA, a cytolysin that is sorted into OMVs produced by pathogenic *E*. *coli*, acts as the targeting component, and is fused to the TEM domain of β-lactamase (Bla), which acts as an enzymatically active probe (Fig 1A), and prevents assembly of the toxin into its biologically active oligomeric conformation [12]. Host cells were incubated with CCF2-AM, a dye composed of a covalently linked coumarin and fluorescein molecule, resulting in FRET and green fluorescence emission, specifically in the eukaryotic cytoplasm where it is processed by esterases. Esterification decreases the hydrophobicity of the FRET probe, thus decreasing its membrane permeability and trapping the probe in the host cell cytoplasm. When OMVs isolated from the producing bacterial strain enter host cells, their Bla cargo is able to cleave CCF2-AM, abolishing FRET and resulting in a shift in emission from green (530 nm) to blue (460 nm) fluorescence (Fig 1A). We monitored the FRET kinetics upon incubation of OMVs with host cells, and analyzed efficiency of OMV uptake by host cells ([Em460/Em530]_t = 0hrs_)/ [Em460/Em530]_t = 3hrs_). We further analyzed data by fitting to a cubic spline function and estimating gradients to extract maximal rate of entry (r_max_) and rate over time (see SI Materials and Methods). Experimental traces were limited to three hours, since beyond this time point the FRET signal decayed, likely due to degradation of the substrate within the host cell cytoplasm.

### Genetically encoded Bla probes are targeted to *E*. *coli* OMVs and retain their enzymatic activity

First, we set out to verify whether ClyA-Bla fusion constructs retained ClyA’s ability to partition into vesicles, and were indeed targeted to *E*. *coli* OMVs. Following induction of probe production, OMVs were isolated from enterohemorrhagic *E*. *coli* (EHEC) containing empty vector, or expressing either ClyA-Bla (C-terminal fusion, enzyme exposed on the OMV surface) or Bla-ClyA (N-terminal fusion, enzyme facing the OMV lumen) enzymatic probes. Probe expression did not significantly change gross OMV morphology (S1A Fig and S1 Text) or charge (mean ζ-potential -6.7 ± 3.6 mV, S1C Fig), but did cause a slight but significant increase in OMV size distribution (~ 20% increase in median diameter; S1B Fig). Probe expression did not appear to result in cell envelope stress, as the amount of OMVs released per cell did not change significantly compared to the untransformed strains (approximately 41 vs 39 vesicles/cell). Sizing data (mean diameter 134 nm, range 10–400 nm across all OMV preparations) were in accordance with previously published data for *E*. *coli* OMVs [12]. Intact ClyA-Bla fusion protein was detected in samples from EHEC whole cell lysate, supernatant and OMV fractions (Fig 1B), suggesting that the fusion protein was targeted to and enriched in OMVs, as previously reported for non-pathogenic *E*. *coli* [12]. The ClyA-Bla probe was oriented with Bla facing the exterior of the OMV, as the protein was gradually degraded during treatment of ClyA-Bla OMVs with papain protease, while the probe remained intact in OMVs containing Bla-ClyA, where Bla faces the vesicle lumen (S1D Fig). The specific enzymatic activity was ~ 3-fold higher for ClyA-Bla OMVs than for Bla-ClyA OMVs with similar activities in whole cell lysates, and both activities were equalized by lysis of vesicles and probe solubilization, suggesting efficient expression of active β-lactamase with the anticipated orientation (inward facing for Bla-ClyA, outward facing for ClyA-Bla) in isolated OMVs (Fig 1C). Average OMV concentration was 5 x 10^12^ particles per ml, and particle concentrations of all samples were normalized to give a consistent OMV concentration for subsequent experiments.

### OMV-targeted Bla probes report on rapid vesicle uptake and dismantling by host cells

**Fig 2:**
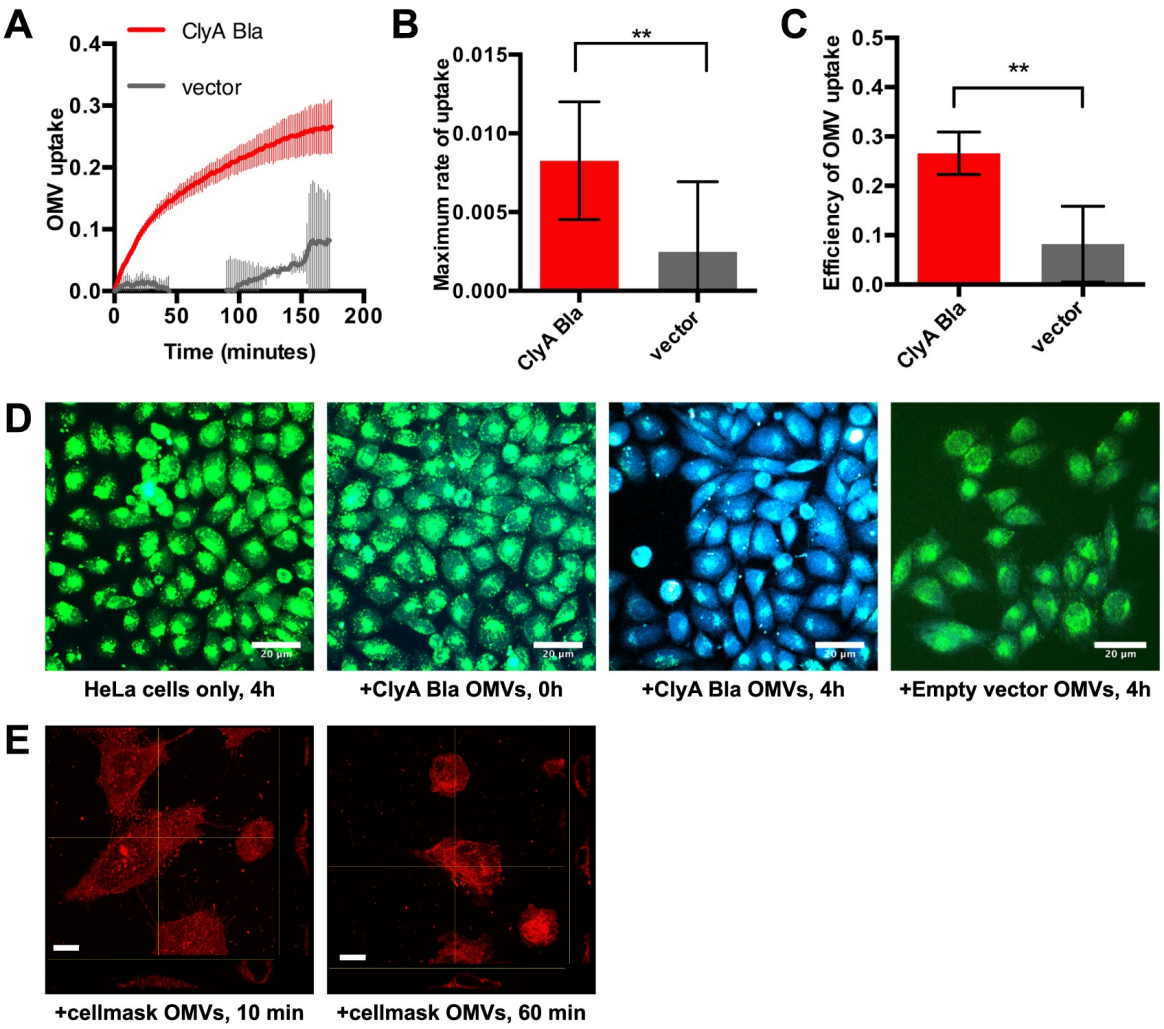
Reporter OMVs capture rapid kinetics of vesicle uptake by host cells in real time. (A) CCF2-AM loaded Hela cells were exposed to OMVs from EHEC carrying ClyA-Bla (red), or vector control (grey) at an MOI of 1000 for 3 hours. Ratio of blue:green fluorescence) over time was plotted as mean ± stdev (n = 3). (B) R_max_ was determined from data in S2A to visualize speed of uptake and is shown are means ± stdev (n = 3). Significance was determined by analysis of variance, with a Brown Forsythe test to determine equal variance. (**) p≤0.01. (C) Absolute FRET changes after 3 h were determined from data in (A) and plotted as efficiency of OMV uptake. Data shown are means ± stdev (n = 3). Significance was determined by ANOVA, with a Brown Forsythe test to determine equal variance. (**) p≤0.01. (D) CCF2-AM loaded Hela cells were imaged by confocal microscopy and merged blue/green images representative of 15 images (n = 3) are shown. Scale bars, 20 μm. (E) Hela cells incubated with cellmask orange-labelled OMVs (red) for 10 and 60 min and slice views of z-stacks were acquired by confocal microscopy. Scale bars, 10 μm.

Having verified the correct targeting, orientation and enzymatic activities of the Bla probes, we used them to dissect OMV entry (i.e., exposure of ClyA-Bla to cytoplasmic dye) and release of OMV luminal contents (i.e., exposure of Bla-ClyA to cytoplasmic dye) into epithelial cells. We used both Hela (cervical epithelial) and RKO (intestinal epithelial) cells loaded with CCF2-AM dye and exposed to OMVs at an MOI of 1000 OMVs/cell. OMV yield was approximately 27 ± 13 OMVs/bacterial cell for the different pathovars used, so this corresponds to a bacterial MOI of approximately 37 bacteria/cell, a dose commonly used in infection assays, or approximately 10 μg/ml OMV protein (published assays use between 5–200 μg/ml OMV protein). EHEC ClyA-Bla OMVs caused a rapid increase in blue/green fluorescence over the course of a 3 hour experiment. OMVs lacking probe did not cause a significant change in FRET signal. (Fig 2A–2C). While the rate of cargo release remains stable throughout the experiment (S2B Fig), the rate of entry is initially high but gradually decreases and approaches the rate of cargo release (S2A Fig). OMV entry kinetics are similar in intestinal epithelial (RKO) cells (S3 Fig). Results of these kinetic analyses were visually confirmed by capturing FRET of samples at the onset and endpoint of the experiment (Fig 2D). The rapid kinetics inferred from the FRET traces also correlated with rapid internalization and re-distribution of OMV lipid inside host cells, with a significant portion of OMV material localized to an intracellular, tubular structure surrounding the nucleus, likely the ER, even after 10 minutes, the fastest we could feasibly prepare samples for imaging (Fig 2E). These results suggest that our approach is capable of capturing the rapid internalization and dismantling of OMVs, which proceeds too fast to adequately capture by imaging. As the rate limiting step for cargo release appears to be OMV entry, we further focused on analyzing potential determinants of the entry process.

### EHEC OMVs enter host cells more rapidly and efficiently than OMVs from non-pathogenic *E*. *coli*

**Fig 3:**
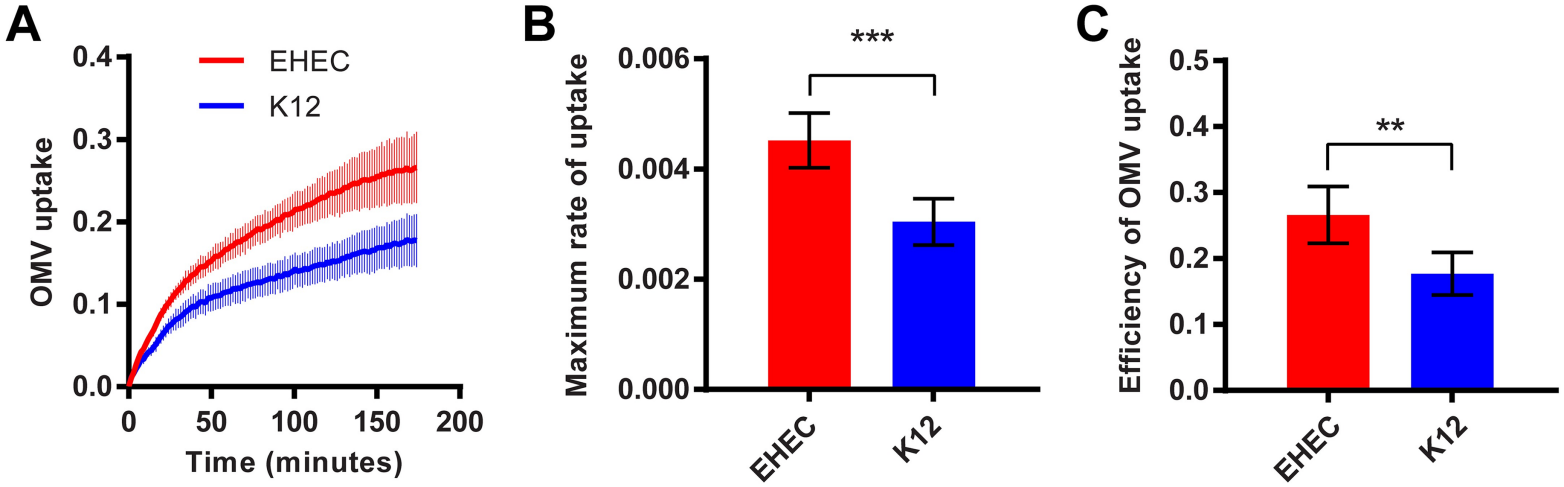
EHEC OMVs enter host cells more rapidly and efficiently than *E*. *coli* K12 OMVs. (A) CCF2-AM loaded Hela cells were exposed to OMVs from EHEC (red) or *E*. *coli* K12 (blue) carrying ClyA-Bla, at an MOI of 1000 for 3 hours. Ratios of blue:green fluorescence over time were plotted as means ± stdev (n = 3). Maximum rates (B) were determined from data in S2 Fig and absolute FRET signal changes after 3 hrs (C) were determined from data in (A) and plotted to visualize overall efficiency of uptake for EHEC (red) and K12 (blue) OMVs. Data shown are means ± stdev (n = 3). Significance was determined by ANOVA, with a Brown Forsythe test to determine equal variance. (***) p≤0.001, (**) p≤0.01.

Next, we compared the uptake kinetics of OMVs isolated from EHEC and non-pathogenic *E*. *coli* K12. Uptake of EHEC OMVs was faster (Fig 3A), and approximately 30% more efficient (Fig 3C), compared to K12 OMVs; the maximal rate was higher (Fig 3B), and a high rate of uptake was sustained for longer for EHEC than for the K12 strain (S2C Fig). Both r_max_ (S2D Fig) and uptake efficiency (S2E Fig) increased with increasing OMV concentration for both EHEC and K12, but for K12 vesicles r_max_ saturated at a lower OMV concentration and a lower uptake efficiency was achieved. Taken together, these results suggest EHEC OMVs contain cargos absent from K12 OMVs that accelerate and sustain the rate and thus increase the efficiency of vesicle uptake by host cells.

### Lipopolysaccharide structure shapes kinetics of OMV uptake by host cells

Since OMVs are derived from the outer membrane of Gram-negative bacteria, they contain lipopolysaccharides (LPS), [23]. Whilst lipid A and the core oligosaccharide regions are well conserved, many species including EHEC contain a highly variable polysaccharide domain known as O antigen [24]. The O antigen constitutes the outermost structural region of LPS, and due to its length of up to 40 nm [24], likely the first component in contact with host cells. These characteristics led us to hypothesize that the O antigen present on EHEC OMVs may be a structural determinant of OMV recognition and uptake by host cells.

**Fig 4:**
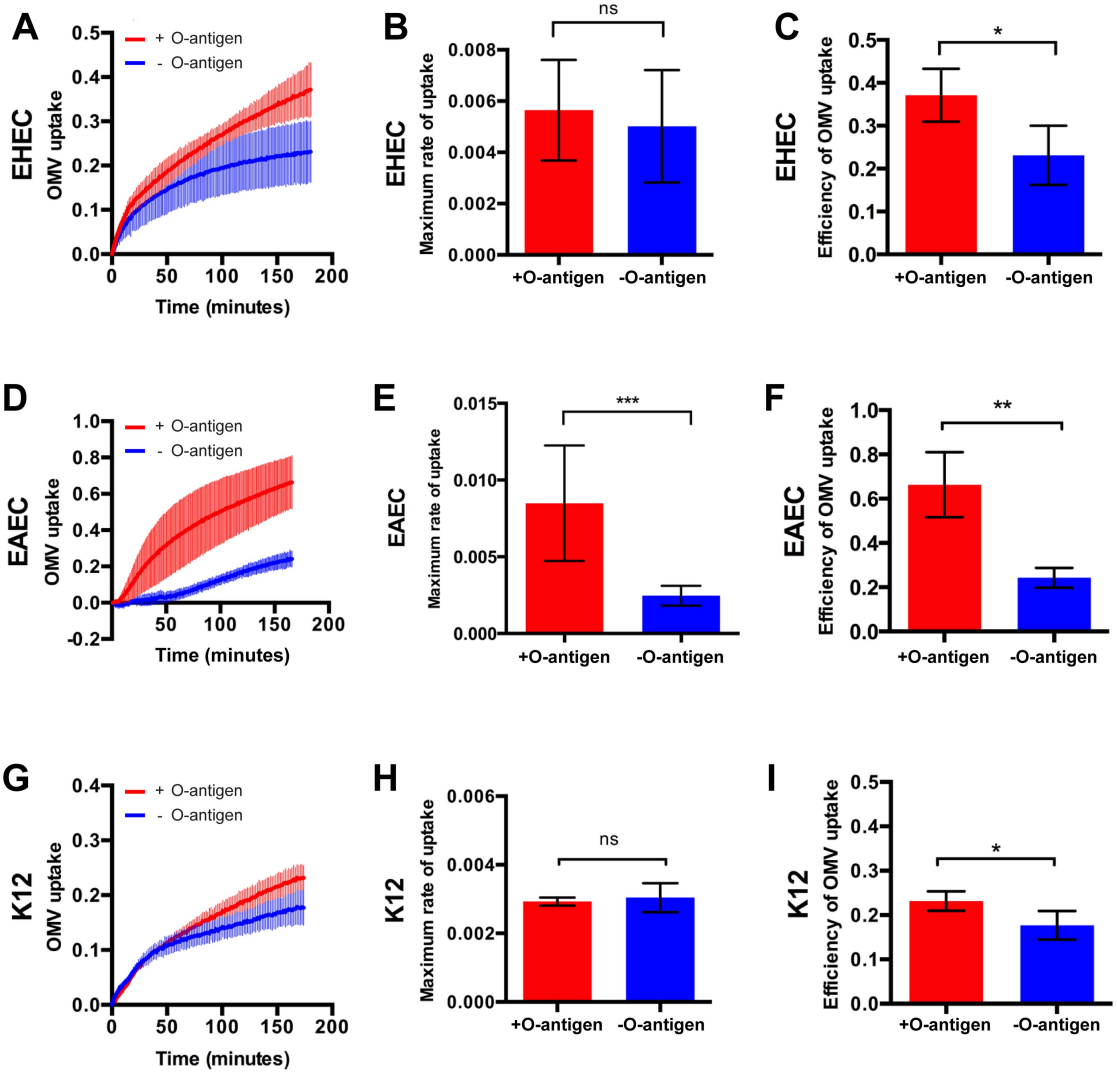
LPS structure affects rate and efficiency of OMV uptake by host cells. CCF2-AM loaded Hela cells were exposed to ClyA-Bla OMVs isolated from EHEC (serotype O157, A-C), EAEC (serotype O42, B-F) or K12 (serotype O16, G-I) containing O antigen (red), or lacking O antigen (blue), at an MOI of 1000 for 3 hours. Ratios of blue:green fluorescence over time (A, D, G) were plotted as means ± stdev (n = 3). Maximum rates (B, E, H) were extracted from data in S4 Fig and absolute FRET changes after 3 hrs (C, F, I) were determined from data shown in A, D and G. Data shown are means ± stdev (n = 3); Significance was determined using ANOVA, with a Brown Forsythe test to determine equal variance. (***) p≤0.001, (**) p≤0.01, (*) p≤0.05, (ns) not significant.

To test this hypothesis, we carried out FRET assays with Hela cells exposed to ClyA-Bla reporter OMVs harvested from three pairs of strains, reflecting different *E*. *coli* serotypes and pathovars and O antigen deficient isogenic mutants, to determine how the presence or absence of O antigen would impact OMV uptake kinetics in each case. OMVs were derived from two different pathovars of *E*. *coli*, EHEC (serotype O157) and enteroaggregative *E*. *coli* (EAEC, serotype O42), and from the non-pathogenic lab strain K12 (serotype O16). For EHEC, OMVs from O157 wild type cells and an isogenic strain lacking the O157 O antigen (*gne*::*IS629*, [25]) were compared (Fig 4). The O antigen deficient mutant *gne*::*IS629* carries a 1310 bp insertion in *gne*, disrupting the epimerase required for synthesis of the oligosaccharide repeating unit in the O antigen [25, 26], leading to a ~ 10 nm decrease in median OMV diameter (S1B Fig). The r_max_ for ClyA-Bla reporter OMVs derived from this O antigen deficient EHEC strain and the isogenic wild type O157 strain were not significantly different (Fig 4B). However, OMVs derived from wild type EHEC with intact O antigen sustained a higher entry rate over a longer period (S4A Fig), and thus entered host cells ~ 43% more efficiently than those derived from O antigen deficient EHEC (Fig 4C).

OMVs from wild type EAEC (serotype O42, intact O antigen) were compared with an isogenic O antigen deficient mutant (Δ*wbaC*, lacking a glycosyltransferase necessary for O antigen synthesis; [27]). EAEC OMVs with intact O antigen were around 20 nm larger in median diameter than EHEC OMVs, suggesting they carry a longer O antigen, and the diameter dropped in the O antigen deficient mutant, to the same size as EHEC O antigen deficient OMVs (S1B Fig). EAEC OMVs with intact O antigen entered host cells ~ 66% more efficiently than OMVs without O antigen, due to a 77% higher r_max_ (Fig 4D–4F) and a higher sustained rate over time (S4B Fig).

The non-pathogenic *E*. *coli* K12 strain MG1655 has lost its ability to produce O antigen due to a disruption in *wbbL* encoding the rhamnosyltransferase required for O antigen synthesis [28]. We compared entry of OMVs from this O antigen deficient strain (median OMV diameter decreased by ~ 10 nm, compared to O16 positive strain), to those from an isogenic strain (DFB 1655 L9), where wild type *wbbL* has been restored, allowing for expression of the strain’s original O16 O antigen [27]. Similar to O157, the presence or absence of O antigen did not alter r_max_, but the presence of O antigen allowed for a higher rate to be sustained for longer (S4C Fig), leading to a ~ 22% higher efficiency overall (Fig 4G–4I). A similar effect of O antigen on uptake kinetics was observed in intestinal epithelial cells (S3 Fig). Taken together, these results suggest that the presence of the LPS O antigen increases the entry efficiency of OMVs into host cells, independent of the specific mutation leading to O antigen deficiency. Depending on the serotype used, this is caused by enhancing r_max_ and/or by sustaining a higher uptake rate over a longer period, compared to OMVs lacking O antigen. These variations may be due to differences in physicochemical features and/or other vesicle cargos between the different serotypes.

### LPS structure determines the preferred entry route of OMVs into host cells

**Fig 5:**
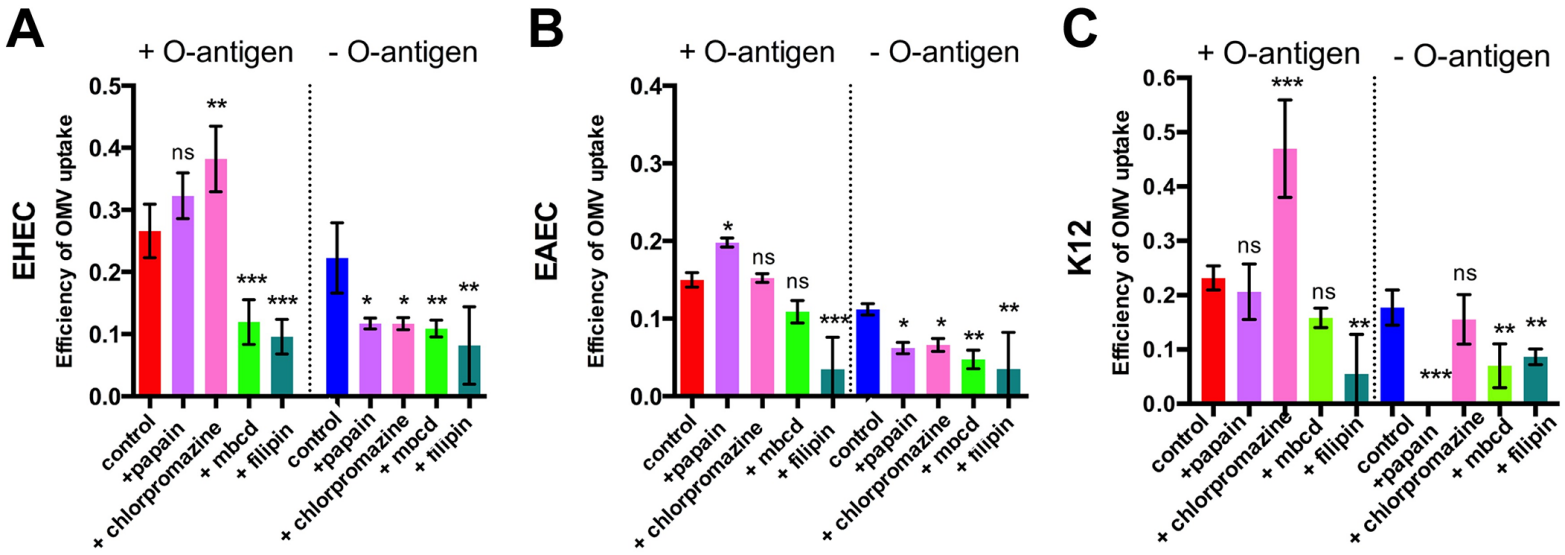
OMVs lacking O antigen are biased towards clathrin-mediated endocytosis, while OMVs with O antigen can efficiently access host cells via lipid rafts. Hela cells were either left untreated (control, red), or pre-treated with 5 μg/ml papain (lilac), 1 μg/ml chlorpromazine (pink), 5 mM methyl-β-cyclodextrin (light green) or 1 μg/ml filipin (turquoise), and exposed to ClyA-Bla OMVs isolated from EHEC (A), EAEC (B) or K12 (C) with or without O antigen at an MOI of 1000 for 3 hours. Total FRET changes after 3 hrs were determined from data in S6 Fig and data shown are means ± stdev (n = 3). Significance compared to the control group was determined using ANOVA, with a Brown Forsythe test to determine equal variance. (***) indicates p≤0.001, (**) p≤0.01, (*) p≤0.05, (ns) not significant.

Next, we evaluated the relative contribution of cellular trafficking pathways to OMV uptake and determined if this was affected by LPS structure. Inhibition of macropinocytosis following treatment of host cells with 20 uM blebbistatin enhanced both the rate and efficiency of uptake in the strains with shorter O antigen (EHEC and K12) and left it unaltered for EAEC (S5 Fig). These data suggest that only a small fraction of OMVs usually enters cells by micropinocytosis, and inhibition of this relatively slow uptake route either does not affect or accelerates uptake. Next, we tested if OMV uptake required dynamin, using the dynamin GTPase inhibitor dynasore. Treatment of host cells with dynasore completely abolished uptake of OMVs, independent of serotype and the presence of O antigen (S5 Fig). Next, we determined whether OMV uptake was via clathrin-coated pits, or via lipid raft-mediated endocytosis, both of which require dynamin [29–31]. We inhibited clathrin-mediated endocytosis, either by proteolytic removal of all protein receptors from host cells with papain prior to OMV incubation, or by blocking pit assembly using chlorpromazine [32]. Removal of protein receptors from the host cell surface increased uptake rate (S6 Fig) and efficiency (Figs 5 and S6) for OMVs with O antigen, but decreased or abolished uptake rate and efficiency of O antigen deficient OMVs. In general, both papain and chlorpromazine treatment decreased the uptake of O antigen negative OMVs but, although they had variable effects, they did not reduce uptake of O antigen positive OMVs (Figs 5 and S6). This suggests that OMVs lacking O antigen require protein receptors for uptake and use clathrin-mediated endocytosis as a main route of entry. In contrast, OMVs with intact O antigen do not rely on protein receptors for entry, and inhibition of clathrin-mediated endocytosis does not prevent their uptake into host cells.

### O antigen containing OMVs enter host cells faster because they can access raft-mediated endocytosis more efficiently

**Fig 6:**
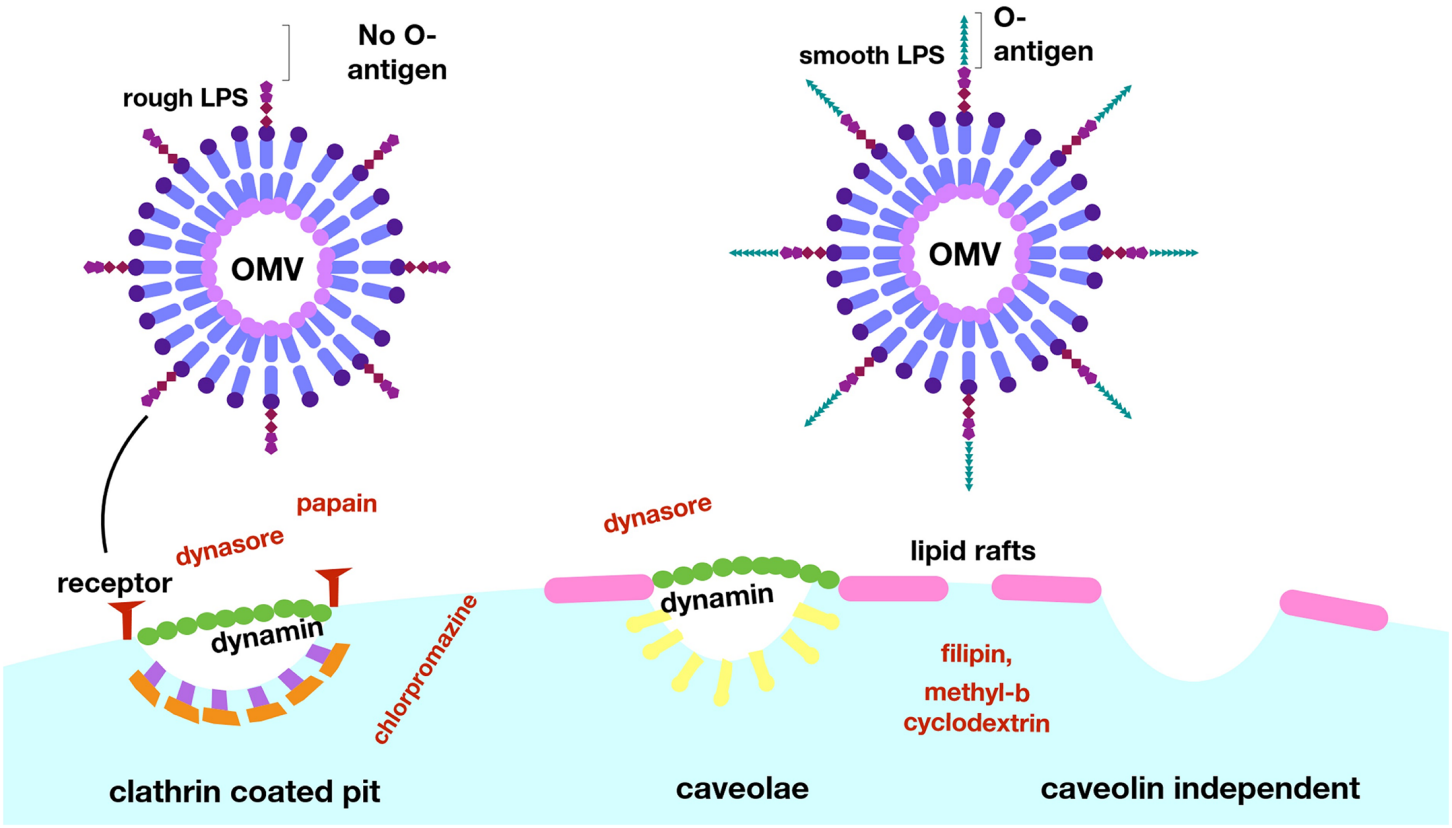
LPS composition determines major route and kinetics of OMV entry into host cells. Whilst it is well established that pathogenic species utilize OMVs during infection, the specific adaptations which allow OMVs to contribute to pathogenesis require further exploration. This work has developed a new approach to overcome current methodological limitations and provide consistent data for future studies and allow new insights into the interactions of OMVs with host cells during infection. This method has shown the relevance of LPS composition, in particular the presence of O antigen, in determining the entry route and kinetics of OMVs. Further work in this area may reveal targets for inhibition of these processes, and enable attenuation of infections by preventing the OMV-associated delivery of virulence factors.

Since OMVs displaying O antigen on their surface accessed host cells faster in the absence of clathrin-dependent endocytosis, we investigated whether this was mediated by raft-dependent pathways. Disruption of raft-mediated endocytosis, either by sequestration of membrane cholesterol from membrane microdomains via methyl-β-cyclodextrin or by disrupting raft dynamics with filipin [33], led to a reduced r_max_ (Fig 5) and uptake efficiency (S6 Fig). These data show that, while OMVs are able to access different uptake routes including macropinocytosis, clathrin-dependent and raft-dependent endocytosis, OMVs displaying O antigen on their surface are able to access raft-dependent endocytosis more efficiently, while OMVs lacking O antigen are more reliant on clathrin-mediated uptake (Fig 6). Shifting a larger fraction of O antigen-positive OMVs to raft-mediated endocytosis further accelerates their uptake, and we conclude the differences in uptake routes driven by LPS structure account for differences in uptake rate and efficiency we observe.

## Discussion

Interactions between bacterial outer membrane vesicles and epithelial cells are now recognized as an important driver of bacterial pathogenesis. Yet, our ability to study vesicle-host cell interactions has been limited by a lack of methods to capture the rapid kinetics of vesicle entry and dismantling in real-time, and without altering the physicochemical properties of the vesicle. Here we describe a novel assay that fulfils these requirements and allowed us to study the kinetics of OMV uptake with enough temporal resolution to reveal critical differences in rate and uptake efficiency of vesicles derived from different *E*. *coli* serotypes and pathovars. The method uses a genetically encoded, OMV targeted probe and a cell-permeable dye, resulting in a change in FRET upon reporter uptake and dye cleavage. Advantages of this system include its high sensitivity (5 μg/ml OMVs, the lowest concentration reported in the literature, produced a reproducible trace with good signal/noise ratio) and rapid response (signal was detected within seconds). A potential drawback is, that it is not known if the ClyA-Bla probe is expressed equally across the entire OMV population, but this is equally true for other markers and assays currently in use. The system’s use can be extended to a high-throughput format, allowing further study of bacterial and host factors determining OMV uptake and trafficking. Using a transwell format, the method can be applied to cell-based assays consisting of bacteria releasing OMVs, and host cells without the need for OMV isolation. Although the specific probes used here were functional across a range of *E*. *coli* isolates and different host cell types, their use in other bacterial species will require further characterization to determine if they are targeted to OMVs and retain correct orientation and enzymatic activity.

We selected EHEC and EAEC OMVs for this study, since OMVs have been shown to play a crucial role in toxin stabilization and delivery for both pathovars [34, 35], and have been considered as a means to vaccinate and protect against hemolytic uremic syndrome, a severe complication of EHEC infection [36]. It is clear that LPS, and specifically O antigen, contributes to bacterial within-host fitness and pathogenicity, by enhancing resistance to complement, modulating phagocytosis and phage infection [37, 38]. The O antigen of most *E*. *coli* strains has 10–18 repeats, but can exceed 80 repeats [39, 40]. The length of the O antigen is equally variable (~5–50 nm), and is positively correlated with the ability of the bacterial cell to adhere to host cells and tissues, while loss of O antigen results in defects in colonisation, biofilm formation, and increased pathogen clearance [24, 41–43]. Recent work showed that EHEC OMVs allow efficient delivery of LPS into the host cell cytoplasm, resulting in inflammatory responses, caspase-11 activation and cell death, but did not explore the role of LPS in uptake [44]. Our data suggest that O antigen has an additional, previously unrecognized role during bacteria-host interactions, which is to steer OMVs towards raft-mediated endocytosis, accelerating uptake and delivery of vesicle associated virulence factors such as hemolysins and Shiga-like toxins [45] to host cells and enhancing pathogenicity.

It is well known that OMVs contain different cargos, depending on pathovar and serotype [46]. This means the comparison of O antigen deficient mutants with wild type OMVs as well as comparison of different pathovars has the pitfall that other vesicle cargos may be modulated and alter uptake kinetics. To dissect the effect of O antigen independent of other cargos, we attempted to deplete O antigen of wild type OMVs by treatment with a glycoside hydrolase, but found enzymatic activity was not limited to O antigen cleavage but modified the core LPS as well. However, we observed a strong correlation between O antigen and uptake kinetics across three different serotypes and pathovars, suggesting that O antigen is, if not the only factor, at least a key determinant of uptake kinetics. Since EAEC OMVs showed the most distinct change in entry kinetics upon O antigen deletion, with r_max_ impacted as well as rate sustenance and efficiency (Fig 4) and O42 antigen seemed to be much longer than EHEC O157 or K12 O16 antigens, which seemed similar in size and displayed similar changes upon O antigen deletion (S1B Fig), we speculate that O antigen length may impact maximal entry rate.

We used our newly-devised assay to identify the relative contribution of cellular uptake pathways to OMV entry into host cells. Clathrin- and raft-dependent endocytosis, macropinocytosis and membrane fusion have all previously been reported as uptake pathways for bacterial OMVs, and it is likely that discrepancies between studies result, at least in part, from differences in species, strains and methodology used to study uptake [47]. Uptake of OMV cargo by fusion of vesicles with the host cell membrane can be ruled out as a major route of uptake for OMVs used in our study, since in this case ClyA-Bla would be exposed on the outer leaflet of the host cell membrane and would not account for the rapid cleavage of the cytoplasmic FRET dye. Assays using pharmacological inhibitors to block specific endocytic pathways, showed that while all OMVs use multiple uptake routes, their surface structure biases them towards different pathways. For example, O antigen deficient OMVs had a stringent requirement for surface protein receptors for their uptake, while O antigen containing OMVs were able to access protein-receptor independent pathways. Depletion of such receptors actually allowed them to access protein-receptor independent pathways more efficiently and utilize raft-mediated endocytosis, a more rapid mode of uptake, as main route of entry. While raft-mediated endocytic routes are not as well characterized as clathrin-mediated endocytosis, it is clear there are multiple pathways, including caveolin and non-caveolin dependent raft-mediated endocytosis. Our experiments suggest that the entry of O antigen containing OMVs is raft- and dynamin dependent, but protein-receptor independent, and no co-localization between OMVs and caveolin was detected. The requirement of dynamin is likely, based on complete inhibition of uptake following treatment with dynasore, however this is confounded by the dual inhibitory effect of dynasore both on dynamin as well as cholesterol containing micro domains [48]. A recent study focusing on vesicular cargo delivery of EHEC OMVs to host cells over longer time frames also concluded that OMVs enter host cells via dynamin-dependent endocytosis [45]. We therefore conclude they use a raft-mediated, and likely dynamin dependent, but protein-receptor and caveolin-independent route of uptake, and the detailed requirements regarding their uptake are subject to current studies.

## Materials and methods

### Strains and growth conditions

The strains used in this study were the *E*. *coli* serotype O157:H7 strain Sakai 813, a derivative of enterohaemorrhagic *E*. *coli* (EHEC) RIMD 0509952, and its O antigen deficient derivative, MA6 (Δ*gne*, [25]; the *E*. *coli* serotype O42 wild type strain (an enteroaggregative *E*. *coli* isolate, [49], and its isogenic, O antigen deficient derivative strain *(*Δ*wbaC*, [27]; the *E*. *coli* serotype O16 strain DFB 1655 L9 (a K12 strain containing a restored *wbbL* gene), and its isogenic, O antigen deficient derivative, MG1655 [27]. All strains were transformed with plasmids pBAD ClyA-Bla, Bla-ClyA, or empty vector (a kan^R^ derivative of the pBAD amp^R^ vector provided by Matthew DeLisa, Cornell University), [12]. Strains were grown in LB containing 50 μg/ml kanamycin, at 37°C with shaking at 200 rpm.

### Isolation of outer membrane vesicles by ultracentrifugation

100 ml cultures were grown in LB at 37°C, with agitation at 200 rpm. Once the OD_600_ reached 0.5–0.6, expression of ClyA-Bla was induced with 0.2% L-arabinose and grown for a further 16 h. Cells were then pelleted at 6000xg, and the supernatants were removed and filtered with a 0.45um syringe filter. Aliquots of filtered supernatants were spread on LB agar and grown overnight at 37°C to check that all viable cells had been removed by filtration. 25 ml of filtered supernatants were centrifuged in a Beckman XL90 ultracentrifuge using a 70Ti rotor at 100,000xg (30,000 rpm) for 2 h at 4°C. After centrifugation, supernatants were removed, and the OMV pellets were resuspended in 1 ml colorless DMEM or sterile water (for TEM) and stored at -20°C.

### Detection of Bla probes in cellular fractions

12 μl of samples normalized for their protein content from EHEC ClyA-Bla and Bla-ClyA whole cell lysate, supernatant and OMV fractions were added to 3μl 5X SDS loading dye and boiled for 10 min. Samples were loaded onto a 15 well BioRad pre-cast stain-free SDS-PAGE gel and run at 120V, 200mA for 45 min. The gel was then transferred onto a PVDF membrane in transfer buffer containing 20% methanol for 80 minutes at 100V. After transfer, the membrane was blocked at room temperature in TBS 0.1% Tween-20 and 5% skim milk for 1h with agitation. The membrane was washed 3 times with TBS 0.1% Tween-20 (5 min per wash). After blocking, the membrane was incubated with a 1:2000 dilution of mouse anti-Bla primary antibody in TBS 0.1% Tween-20 and 5% skim milk overnight at 4°C with agitation. The following day, the membrane was washed 3 times as before, and incubated with a 1:5000 dilution of sheep anti-mouse secondary antibody in TBS 0.1% Tween-20, 5% skim milk for 1h at room temperature with agitation. The membrane was washed again 3 times, and 2 ml BioRad ECL reagents were added to the membrane and incubated for 5 min, before visualization with a BioRad ChemiDoc imager.

### Nitrocefin assay to determine β-lactamase activity

50 μl of samples were added in triplicate to a 96-well plate. Nitrocefin was diluted to 0.5 mg/ml in PBS and 50 μl was added to each sample. The absorbance at 486 nm was measured in the FluoStar Omega plate reader for 2 h, and the change in absorbance over time was used to determine the specific activity in samples, using the protein concentration determined by the CBQCA kit.

### Protein quantitation

To quantify levels of protein in cell fractions, the ThermoFisher CBQCA Protein Quantitation kit was used according to the manufacturer’s instructions.

### Papain and detergent treatment of OMVs

Triton X-100 and SDS were added at a concentration of 1% to 20 μl OMVs for 45 min at 37°C. 5ug/ml papain was then added for 30 or 60 min at 37°C. The papain reaction was inactivated using 1 mM PMSF at room temperature for 30 min. 5 μl SDS-PAGE loading dye was added to the samples, which were then boiled for 10 min. Samples were run on a 15-well pre-cast stain free gel for 45 min at 120V, and then subjected to Western blotting with anti-β-lactamase primary antibody (Pierce) as described above.

### Plate reader FRET experiments

HeLa cells (passage 1–7) were seeded in triplicate in a black-walled, clear bottom 96-well plate at a concentration of 1x10^5^ cells per ml in Dulbecco’s modified Eagle medium (DMEM) supplemented with 1% L-glutamine, 1% Penicillin/Streptomycin and 10% heat inactivated fetal bovine serum. The plate was incubated at 37°C, 5% CO_2_ for 24 h prior to experiments. The following day, cells were loaded with 20 μl 6X CCF2-AM with 100 μl colourless unsupplemented DMEM (cDMEM) and incubated at room temperature for 1 h in the dark to allow dye loading. The dye was removed by washing 2x in PBS and 1x in cDMEM. Cells were treated with 5 mM methyl-ß-cyclodextrin or 1 μg/ml filipin to inhibit cholesterol mediated endocytosis, 80 uM Dynasore for dynamin inhibition, or 20 uM blebbistatin for macropinocytosis inhibition for 1h at 37°C. Cells were treated with 1 μg/ml chlorpromazine for 1h at 37°C to inhibit formation of clathrin-coated pits, or with 5 μg/ml papain for 15 min at 37°C to remove surface proteins, before inactivation of papain with 5 mM PMSF for 20 min.

Reporter OMVs were diluted in cDMEM and added to the cells for a final concentration of 10 μg/ml, or 1x10^8^ vesicles, corresponding to an MOI of 1000. The plate was immediately placed in the PheraStar plate reader, with excitation at 405 nm and simultaneous dual emission at 530 nm and 460 nm. The wells were scanned (bottom optic) with orbital averaging for a total of 150 cycles, equating to a measurement every 90 seconds for 3 hours. The ratio of blue to green fluorescence intensity detected in the cells at each cycle was calculated using GraphPad Prism, and ratios for uninfected, dye-loaded cells were used as the baseline value for each cycle. All traces were normalized to 0 for their first ratio value. All experiments were performed with a minimum of three technical replicates and three independent repeats.

### Efficiency of uptake and statistical analysis

Efficiency of uptake was calculated as the absolute change in blue:green fluorescence intensity ratio between 0 and 3 hours ([Em460/Em530]_t = 0hrs_)/ [Em460/Em530]_t = 3hrs_). Analysis of variance (ANOVA) was used to determine statistical significance, with a Brown Forsythe test to determine equal variance (GraphPad Prism software). A p-value of <0.05 was considered statistically significant.

### Rate estimation and statistical analysis

To estimate the gradients of the data, polynomials were fitted to each data set using the cubic spline function *csaps* in Matlab. Numerical estimates of the gradients of the resulting polynomials were determined using the *gradient* function. To ensure that the gradient estimates were as smooth as possible whilst also retaining the overall shape and trend of the data, a small smoothing parameter was used. Analysis of variance (ANOVA) was used to determine statistical significance, with a Brown Forsythe test to determine equal variance (GraphPad Prism software). A p-value of <0.05 was considered statistically significant.

### Confocal microscopy

HeLa cells (P3-7) were seeded on 13mm coverslips in a 12-well plate at a concentration of 1x10^5^ cells per ml in complete DMEM, 24 h prior to experiments. The following day, cells were washed and loaded with 100 μl 6X CCF2-AM dye with 500 μl colourless unsupplemented DMEM, and incubated in the dye solution for 1 h at room temperature in the dark. Cells were then incubated with ClyA-Bla reporter OMVs for 0–4 h. The cells were washed with PBS and then fixed with 0.5 ml 4% PFA. The next day, coverslips were mounted onto slides with a drop of Gold Anti-Fade mounting solution and then imaged using a Nikon A1R confocal microscope (Birmingham Advanced Light Microscopy Facility), and fluorescence was observed from excitation at 409 nm and dual emissions at 450 nm and 520 nm. Z stacks were produced with gain, slice thickness, exposure and laser intensity kept the same for all slides, and images were taken for 3 representative fields of view per slide and n = 3 independent samples. The Z stacks were converted to maximum intensity projection images. For OMV localization experiments, OMVs were stained using cell mask orange (1:500) for 1 h at 22°C and gentle agitation. Following staining, samples were washed with 28 volumes of PBS and labelled OMVs pelleted by ultracentrifugation (100,000xg, 2h). Hela cells were exposed to labelled OMVs for 10 of 60 minutes prior to fixation in 3.2% formaldehyde. Slides were imaged using an Olympus IX83 inverted microscope fitted with a FV3000 confocal system and 100x Super Apochromat oil objective. Images were captured using Olympus Fluoview software and processed using the CellSens extension package.

## Supporting information

S1 Text Description of supporting materials and methods. (DOCX)

S1 Fig Morphology, size, charge and probe orientation of reporter OMVs. (A) Electron micrographs of negative stained OMV fractions from EHEC wt (left image) or EHEC ClyA-Bla (centre and right images). Scale bars, 0.5 μm. (B) Isolated OMVs were diluted 1x10^-6^ fold and nanoparticle tracking analysis was used to determine the size distribution. Black lines represents median size from at least 200 tracks acquired per sample. Statistical significance was determined by ANOVA, with a Brown Forsythe test to determine equal variance. (***) p≤0.005, (ns) not significant. (C) ζ-potentials of isolated OMVs. Values represent means from 30 readings per sample. Only means are displayed since individual readings are not accessible instrumentally. (D) OMV fractions from EHEC expressing Cly-Bla, Bla-ClyA or carrying empty vector were treated with papain for 30 or 60 minutes, and used for Western Blotting with α-Bla antibody.
(TIF)

S2 Fig Rates of uptake/dismantling and concentration dependency of uptake kinetics for OMVs. (A) CCF2-AM loaded Hela cells exposed to EHEC OMVs carrying ClyA-Bla (red), or empty vector (grey) at an MOI of 1000 for 3 h. Rate of uptake over time was extracted from data in Fig 2A and data shown are means ± stdev (n = 3). (B) FRET change upon exposure of Hela cells to EHEC OMVs carrying ClyA-Bla (reporting on exposure to OMV surface to cytoplasm) or Bla-ClyA (reporting on exposure of luminal cargo to cytoplasm). (C) Hela cells were exposed to EHEC or K12 ClyA-Bla OMVs at an MOI of 1000 for 3 hours. Rates of uptake over time were extracted from data in Fig 3A and are means ± stdev (n = 3). (D) Experiments were repeated as above but using different OMV concentrations (0–20 μg/ml of protein, corresponding to an MOI of 0–2000), and maximum rates (D) and efficiency of uptake (E) determined as described above. Data are means ± stdev (n = 3).
(TIF)

S3 Fig Uptake for OMVs from serotypes O157, O42 and O16 with or without O antigen. CCF2-AM loaded RKO intestinal epithelial cells were exposed to OMVs from EHEC O157 (A), EAEC O42 (B), and K12 O16 (C), with O antigen (red) and without O antigen (blue), at an MOI of 1000 for 3 hours. FRET changes (blue/green fluorescence, A-C) and efficiency of uptake (total change over three hours, D) are shown as means ± stdev (n = 3).
(TIF)

S4 Fig Rates of uptake for OMVs from serotypes O157, O42 and O16 with or without O antigen. CCF2-AM loaded Hela cells were exposed to OMVs from EHEC O157 (A), EAEC O42 (B), and K12 O16 (C), with O antigen (red) and without O antigen (blue), at an MOI of 1000 for 3 hours. Polynomials were fitted to each data set using the cubic spline function csaps in Matlab. Numerical estimates of the gradients of the resulting polynomials were determined using the gradient function. Data shown are means ± stdev (n = 3).
(TIF)

S5 Fig Effect of blebbistatin and dynasore on uptake of OMVs. Hela cells were either left untreated or pre-treated 80 uM Dynasore for dynamin inhibition (grey), or 20 uM blebbistatin for macropinocytosis inhibition (orange) for 1h at 37°C and exposed to ClyA-Bla OMVs isolated from EHEC (A, B), EAEC (C, D), or K12 (E, F) at an MOI of 1000 for 3 hours. The FRET signal (ratio of blue:green fluorescence) over time was plotted as mean ± stdev (n = 3).
(TIF)

S6 Fig Effect of pharmacological treatments on OMV uptake. Hela cells were either left untreated or pre-treated with 5 ug/ml papain (lilac), 1 ug/ml chlorpromazine (pink), 5mM methyl-β-cyclodextrin (light green) or 1μg/ml filipin (turquoise) and exposed to ClyA-Bla OMVs isolated from EHEC (A, B), EAEC (C, D), or K12 (E, F) at an MOI of 1000 for 3 hours. The FRET signal (ratio of blue:green fluorescence) over time was plotted as means ± stdev (n = 3).
(TIF)
