# Supplementary material for: Lipopolysaccharide structure impacts the entry kinetics of bacterial outer membrane vesicles into host cells
Source: PLoS Pathog. 2017 Nov 29;13(11):e1006760. doi: 10.1371/journal.ppat.1006760 (PMC5724897; doi:10.1371/journal.ppat.1006760)
Supplement: S1 Text — (DOCX) [file ppat.1006760.s001.docx]

**SUPPORTING MATERIALS AND METHODS**

**Nanoparticle tracking analysis**

After purification, OMV samples were diluted 1x10^-6^ in filtered sterile PBS. Particle diameter and concentration were measured using the Nanosight LM10 particle tracking analysis, with a minimum of 100 tracks per sample, performed in triplicate. Camera shutter 1495 and gain of 450 were used, and size distribution scatter plots were created using GraphPad Prism. Size distribution was analysed using analysis of variance (ANOVA) with a Brown Forsythe test for equal variance.

**Measurement of ζ-potential as an indicator of OMV surface charge**

700 µl of OMV preparations were analysed using a Zeta Sizer (Malvern Instruments) and data from an average of 30 readings/sample were acquired at 37 °C and means were plotted.

**Visualization of outer membrane vesicles by Transmission Electron Microscopy**

10μl of isolated outer membrane vesicles in sterile deionized distilled water were added to 400-mesh copper grids, and negatively stained with 4% uranyl acetate for 2 min. Samples were then observed using a Jeol 1200Ex transmission electron microscope (Birmingham Electron Microscopy Facility) with an acceleration of 75kV.
