# Supplementary figures and images for: Lipopolysaccharide structure impacts the entry kinetics of bacterial outer membrane vesicles into host cells
Source: PLoS Pathog. 2017 Nov 29;13(11):e1006760. doi: 10.1371/journal.ppat.1006760 (PMC5724897; doi:10.1371/journal.ppat.1006760)

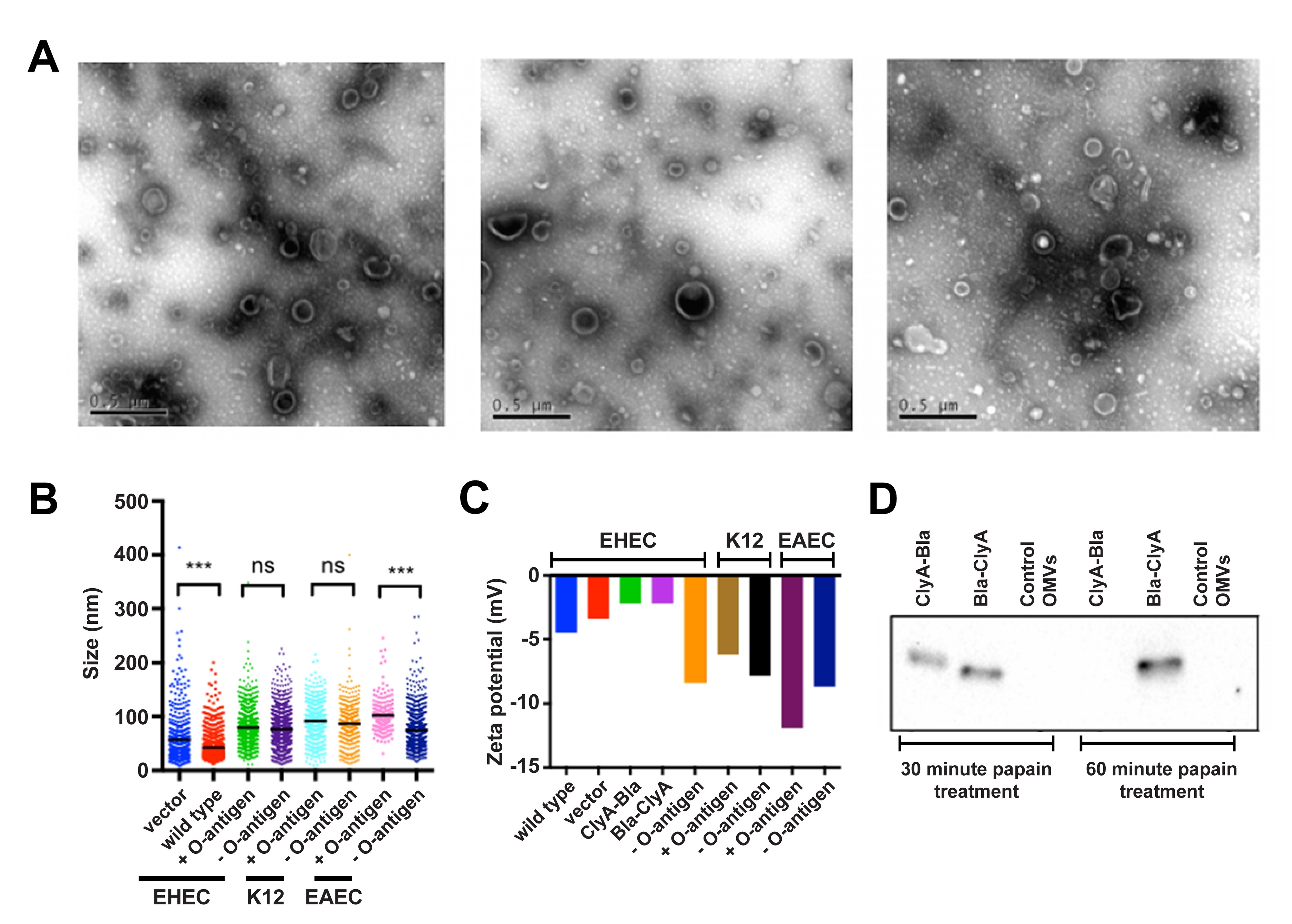

Supplement: S1 Fig — (A) Electron micrographs of negative stained OMV fractions from EHEC wt (left image) or EHEC ClyA-Bla (centre and right images). Scale bars, 0.5 μm. (B) Isolated OMVs were diluted 1x10^-6^ fold and nanoparticle tracking analysis was used to determine the size distribution. Black lines represents median size from at least 200 tracks acquired per sample. Statistical significance was determined by ANOVA, with a Brown Forsythe test to determine equal variance. (***) p≤0.005, (ns) not significant. (C) ζ-potentials of isolated OMVs. Values represent means from 30 readings per sample. Only means are displayed since individual readings are not accessible instrumentally. (D) OMV fractions from EHEC expressing Cly-Bla, Bla-ClyA or carrying empty vector were treated with papain for 30 or 60 minutes, and used for Western Blotting with α-Bla antibody. (TIF) [file ppat.1006760.s002.tif]

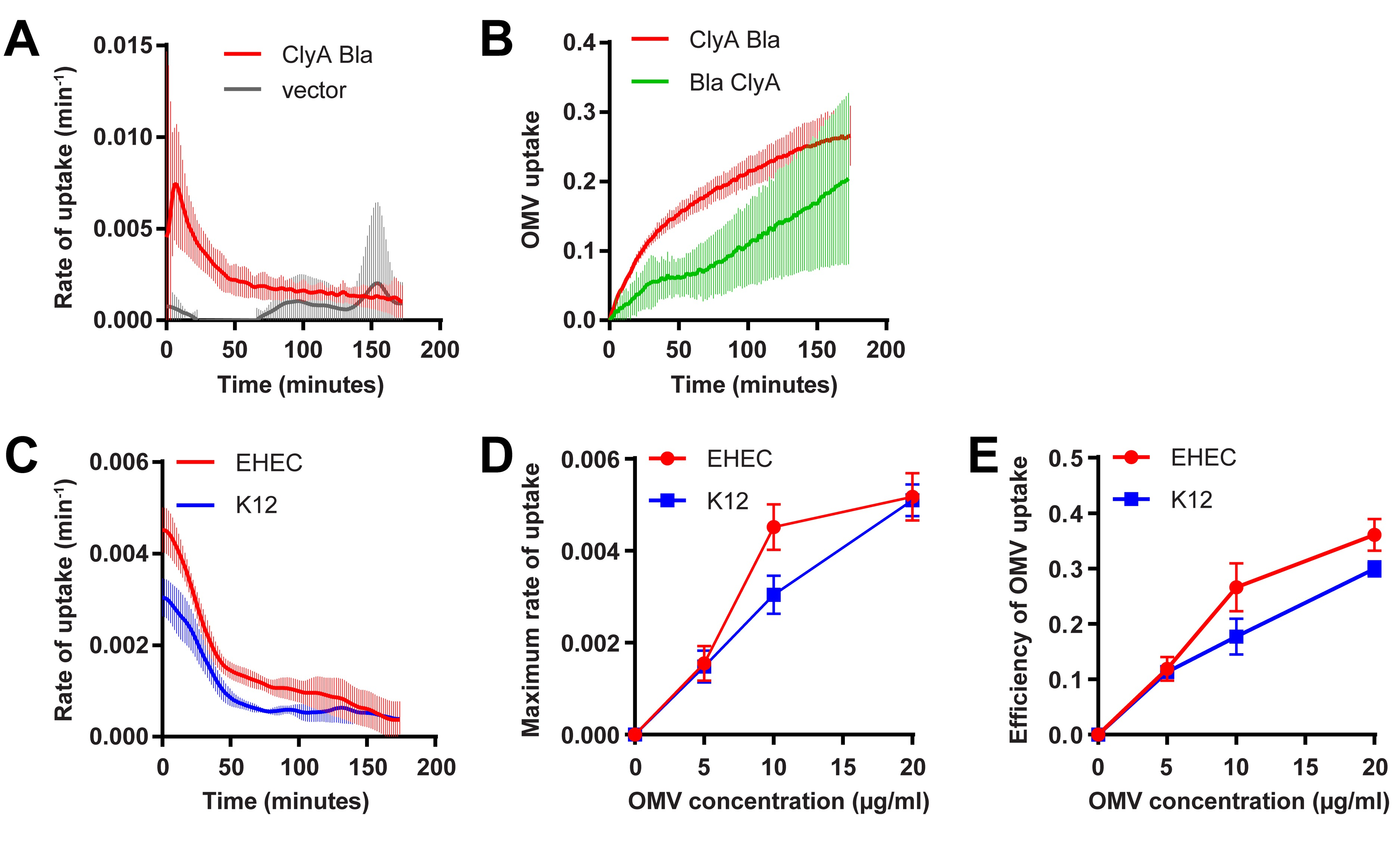

Supplement: S2 Fig — (A) CCF2-AM loaded Hela cells exposed to EHEC OMVs carrying ClyA-Bla (red), or empty vector (grey) at an MOI of 1000 for 3 h. Rate of uptake over time was extracted from data in Fig 2A and data shown are means ± stdev (n = 3). (B) FRET change upon exposure of Hela cells to EHEC OMVs carrying ClyA-Bla (reporting on exposure to OMV surface to cytoplasm) or Bla-ClyA (reporting on exposure of luminal cargo to cytoplasm). (C) Hela cells were exposed to EHEC or K12 ClyA-Bla OMVs at an MOI of 1000 for 3 hours. Rates of uptake over time were extracted from data in Fig 3A and are means ± stdev (n = 3). (D) Experiments were repeated as above but using different OMV concentrations (0–20 μg/ml of protein, corresponding to an MOI of 0–2000), and maximum rates (D) and efficiency of uptake (E) determined as described above. Data are means ± stdev (n = 3). (TIF) [file ppat.1006760.s003.tif]

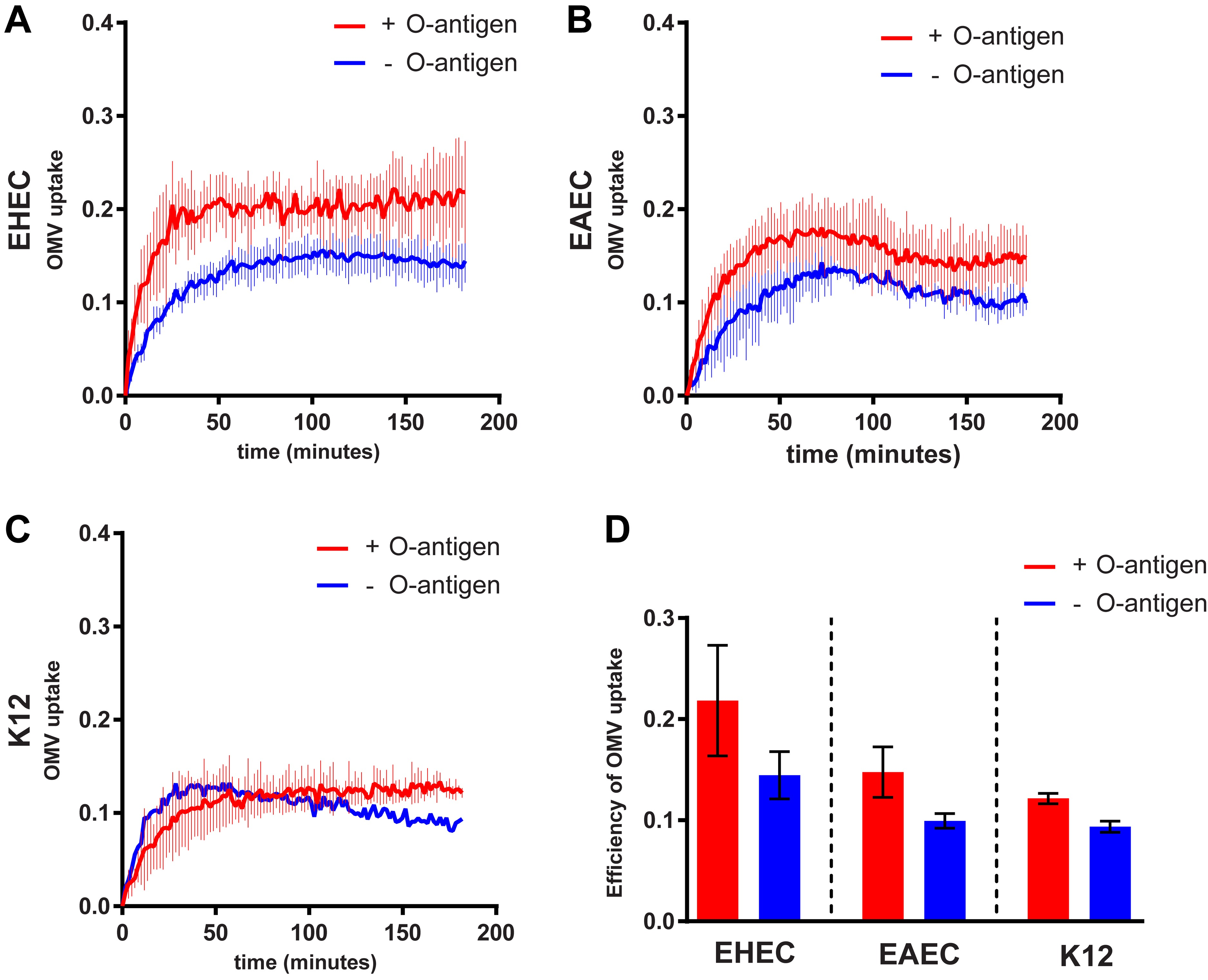

Supplement: S3 Fig — CCF2-AM loaded RKO intestinal epithelial cells were exposed to OMVs from EHEC O157 (A), EAEC O42 (B), and K12 O16 (C), with O antigen (red) and without O antigen (blue), at an MOI of 1000 for 3 hours. FRET changes (blue/green fluorescence, A-C) and efficiency of uptake (total change over three hours, D) are shown as means ± stdev (n = 3). (TIF) [file ppat.1006760.s004.tif]

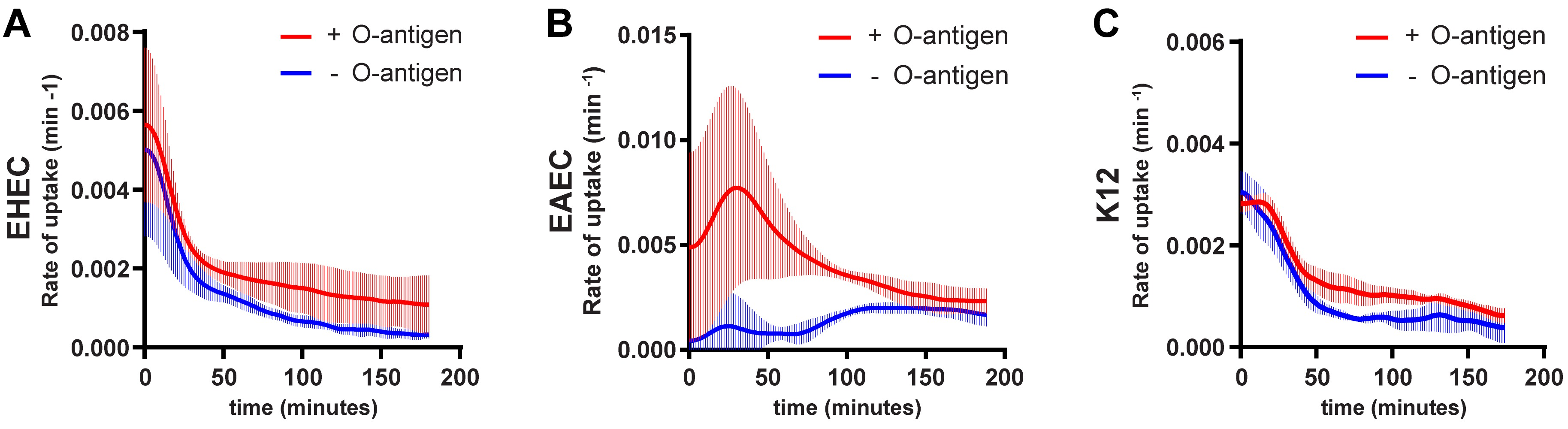

Supplement: S4 Fig — CCF2-AM loaded Hela cells were exposed to OMVs from EHEC O157 (A), EAEC O42 (B), and K12 O16 (C), with O antigen (red) and without O antigen (blue), at an MOI of 1000 for 3 hours. Polynomials were fitted to each data set using the cubic spline function csaps in Matlab. Numerical estimates of the gradients of the resulting polynomials were determined using the gradient function. Data shown are means ± stdev (n = 3). (TIF) [file ppat.1006760.s005.tif]

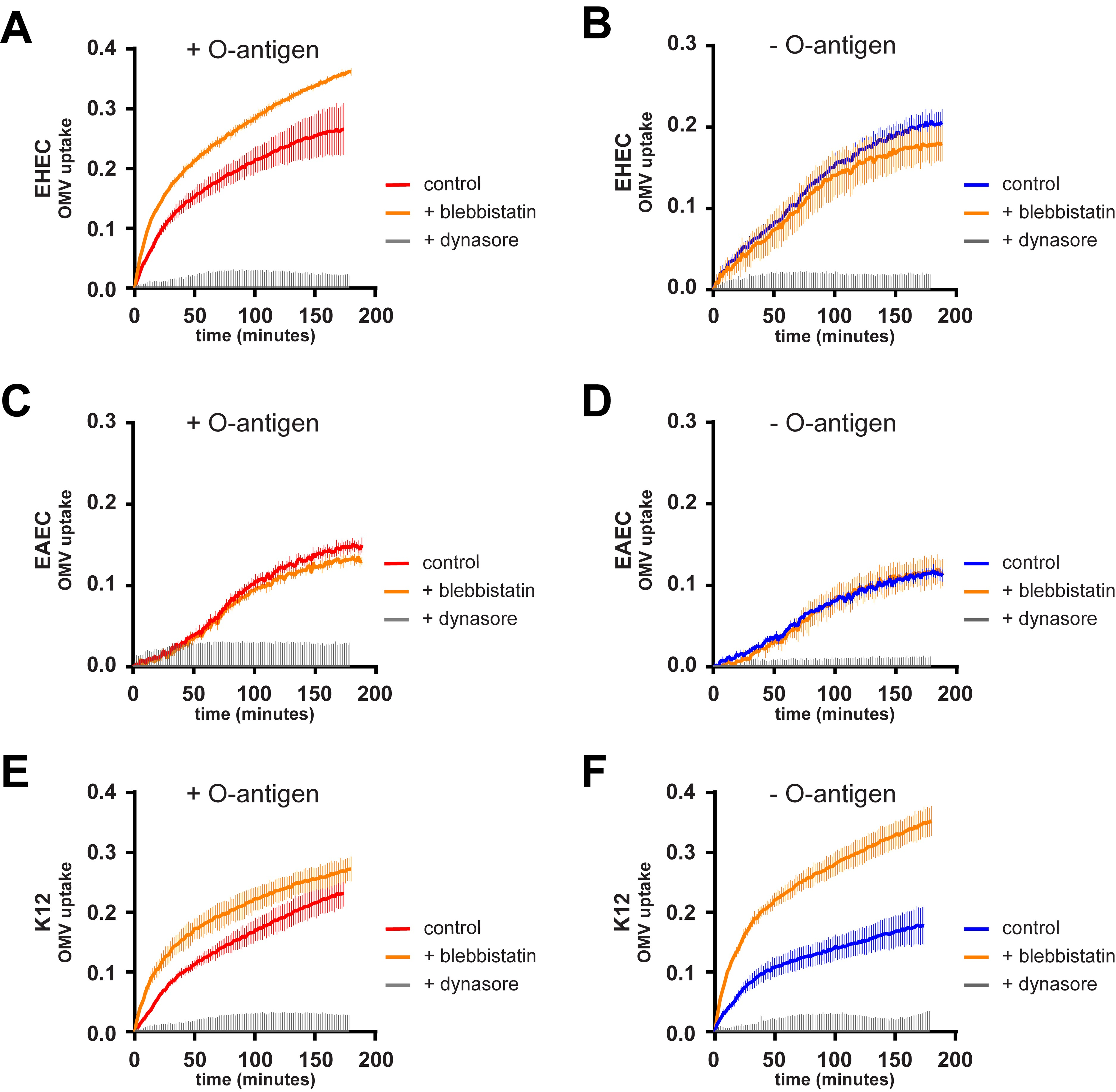

Supplement: S5 Fig — Hela cells were either left untreated or pre-treated 80 uM Dynasore for dynamin inhibition (grey), or 20 uM blebbistatin for macropinocytosis inhibition (orange) for 1h at 37°C and exposed to ClyA-Bla OMVs isolated from EHEC (A, B), EAEC (C, D), or K12 (E, F) at an MOI of 1000 for 3 hours. The FRET signal (ratio of blue:green fluorescence) over time was plotted as mean ± stdev (n = 3). (TIF) [file ppat.1006760.s006.tif]

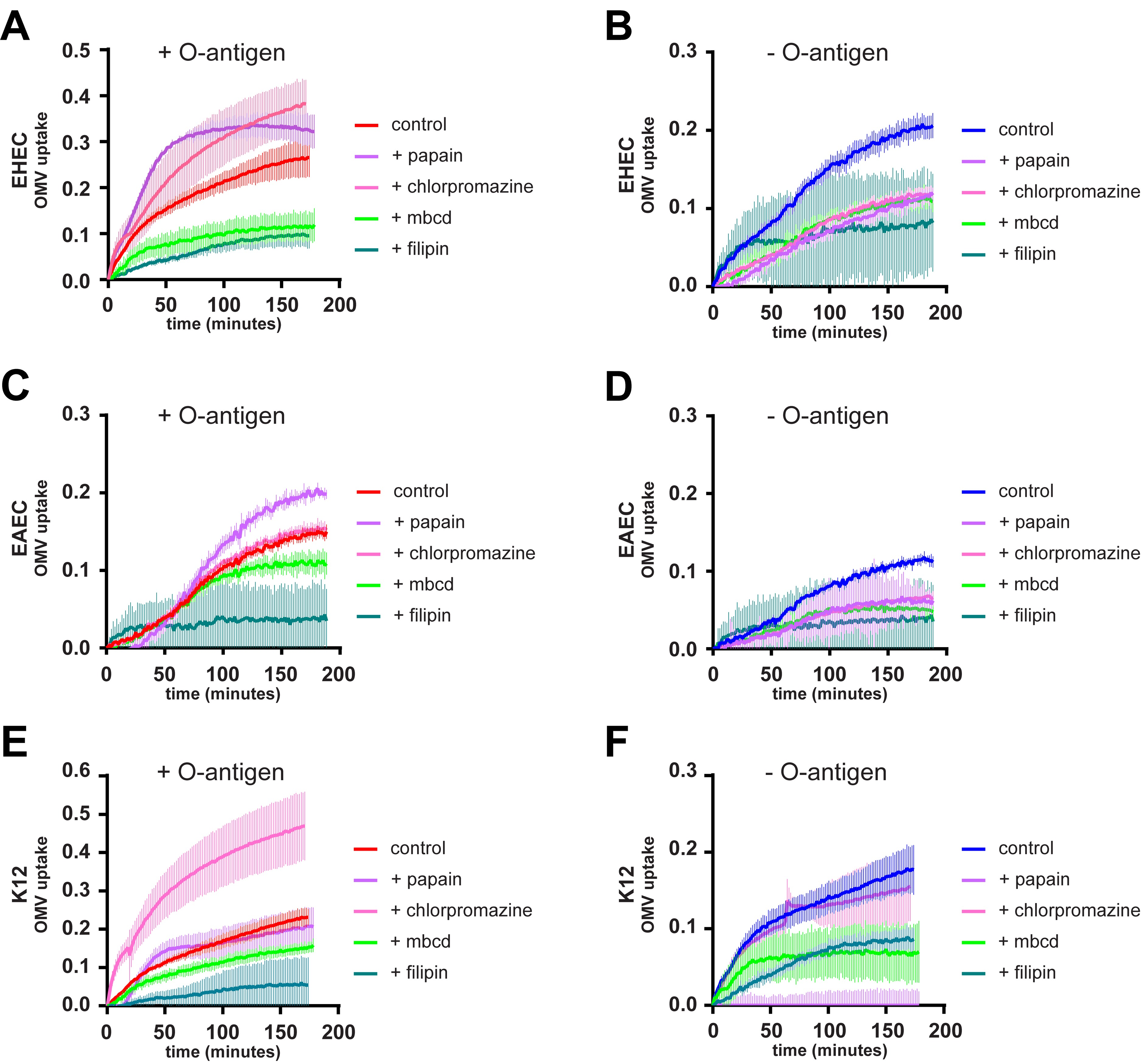

Supplement: S6 Fig — Hela cells were either left untreated or pre-treated with 5 ug/ml papain (lilac), 1 ug/ml chlorpromazine (pink), 5mM methyl-β-cyclodextrin (light green) or 1μg/ml filipin (turquoise) and exposed to ClyA-Bla OMVs isolated from EHEC (A, B), EAEC (C, D), or K12 (E, F) at an MOI of 1000 for 3 hours. The FRET signal (ratio of blue:green fluorescence) over time was plotted as means ± stdev (n = 3). (TIF) [file ppat.1006760.s007.tif]
